# Dyes Confinement in the Nano Scale and Converting Poly Vinyl Alcohol to Be Optical-Active Polymeric Nanocomposites with High Thermal Stability

**DOI:** 10.3390/polym15102310

**Published:** 2023-05-15

**Authors:** Adil Alshoaibi

**Affiliations:** Department of Physics, College of Science, King Faisal University, Al-Ahsa 31982, Saudi Arabia; adshoaibi@kfu.edu.sa

**Keywords:** Zn-Al-green dyes nanohybrids, PVA-Zn-Al dyes nanocomposite, UV–Vis absorption spectra, band gap, scanning electron microscopy, thermal analyses

## Abstract

In the present research, groups of nanolayered structures and nanohybrids based on organic green dyes and inorganic species are designated to act as fillers for PVA to induce new optical sites and increase its thermal stability through producing polymeric nanocomposites. In this trend, different percentages of naphthol green B were intercalated as pillars inside the Zn-Al nanolayered structures to form green organic-inorganic nanohybrids. The two-dimensional green nanohybrids were identified by X-ray diffraction, TEM and SEM. According to the thermal analyses, the nanohybrid, which has the highest amount of green dyes, was used for modifying the PVA through two series. In the first series, three nanocomposites were prepared depending on the green nanohybrid as prepared. In the second series, the yellow nanohybrid, which was produced from the green nanohybrid by thermal treatment, was used to produce another three nanocomposites. The optical properties revealed that the polymeric nanocomposites depending on green nanohybrids became optical-active in UV and visible regions because the energy band gap decreased to 2.2 eV. In addition, the energy band gap of the nanocomposites which depended on yellow nanohybrids was 2.5 eV. The thermal analyses indicated that the polymeric nanocomposites are thermally more stable than that of the original PVA. Finally, the dual functionality of organic-inorganic nanohybrids that were produced from the confinement of organic dyes and the thermal stability of inorganic species converted the non-optical PVA to optical-active polymer in a wide range with high thermal stability.

## 1. Introduction

The low thermal stability of polymers is due to their hydrocarbon structures. Therefore, this can be improved through combining with inorganic materials that have high thermal stability. Actually, inorganic materials can be employed to decrease the thermal degradation rate or increase the heat resistance [1,2,3,4]. In order to improve the optical properties of polymers, dyes are good candidates for creating new optical centers inside the structure of polymers. In this trend, to improve the thermal properties and optical activities of polymers, the incorporation of inorganic particles or layers in the nano scale inside polymers is considered an efficient technique for increasing their applications. However, the incompatibility between organic polymer and inorganic species leads to a phase separation. This challenge, to obtain thermodynamically stable hybrids, can be achieved through dispersing the inorganic nanomaterials inside the polymer in a homogenous state. In order to obtain a high degree of dispersion inside polymers, the inorganic nanomaterials are covered or anchored by favorable organic compounds. These compounds have hydrophobic characters to be compatible with polymers and useful for many applications. 

The astonishing physical and chemical properties of organic-inorganic nanohybrids [5,6,7,8,9,10,11] have strongly attracted the attention of the scientific societies because conventional processes cannot lead to these properties. The combination between organic and inorganic compounds in nanostructures can create unlimited groups of novel compounds through producing a wide range of unknown and known activities. In this trend, to improve the thermal properties and optical activities of polymers, the incorporation of inorganic particles or layers in the nano scale inside polymers is considered an efficient technique for increasing their applications. However, the incompatibility between organic polymers and inorganic species leads to a phase separation. This challenge, to obtain thermodynamically stable hybrids, can be achieved through dispersing the inorganic nanomaterials inside the polymer in a homogenous state. In order to obtain a high degree of dispersion inside polymers, the inorganic nanomaterials are covered or anchored by favorable organic compounds. These compounds have hydrophobic characters to be compatible with polymers and useful for many applications.

PVA has been largely studied because of the mechanical properties, biocompatibility and chemical resistance [12,13]. Therefore, it was suitable for many industrial products such as paints, adhesives, coatings and membranes [14]. Recently, PVA has used in the biomedical applications for artificial organs, contact lenses [15,16], drug delivery systems [17,18], engineering of tissues [19] and dialysis membrane [20]. These wide applications directed the attention of researchers for developing the optical properties of PVA and improving its thermal stability to become more favorable and promising for new applications.

Mallakpour and Dinari [21] have used alumina nanoparticles as filler for increasing the thermal stability of PVA. In another study, titanium oxide nanoparticles were used as dopant for PVA to increase its mechanical properties [22]. Du et al. have used PVA for increasing capacitance performance through designing polymeric electrodes [23]. Abd-Elrahman reported that the thermal stability PVA increased by producing composite structures with zinc oxide [24]. Farrag et al. indicated that the optical properties of PVA increased through narrowing the band gap from 4.4 eV to 2.9 eV by combining with multi walls carbon nanotubes [25]. Peranidze et al. have successfully used the composites of PVA for bone tissue engineering [26].

To create new optical sites and increase the thermal properties of poly (vinyl alcohol), new nanohybrids can be designed depending on confinement of green dyes of naphthol green B in the nano scale among inorganic nanolayered structures. 

Among inorganic layered structures, there is a group of two-dimensional nanostructured materials calls layered double hydroxides (LDHs). This kind of nanolayered materials consists mainly of inorganic species [27,28,29,30,31] and has the ability to combine with organic compounds as interlayered anions through host–guest interactions. The host is LDHs composing of di-and tri-valent elements in an order arrangement and their nanolayers are cationic in nature. This cationic nature of the nanolayers is neutralized by organic anions that are used as guests among the layers in a confined state. The confined guests among the nanolayers of two-dimensional structures have only one dimension orientation. This confinement is not rigid because the size and orientation of guests control the interlayered spacing between the inorganic nanolayers producing hybrid lamellae. These hybrid lamellae that have widths of 0.1–1 µm and thickness of 0.5–1 nm are suitable fillers with a large aspect ratio and are able to perform intercalation reactions with polymers. By controlling the organic and inorganic species of these hybrid lamellae, the dispersion of the lamellae inside polymers produces polymeric nanocomposites and increases their applications in the different fields.

In the current study, polymeric nanocomposites that are composed of inorganic and organic dye species in addition to polymers have attracted our attention because of the multi-functionality of these hybrids. To become PVA, optically active in the UV region and visible region in addition to increasing its thermal stability, new nanohybrids based on green dyes of naphthol green B were prepared through host–guest interaction with Zn-Al LDHs. In addition, a new series of nanoparticles of multi-oxides doped with dyes was produced through thermal treatment. Using these nanohybrids, which are used as fillers for PVA, the produced polymeric nanocomposites have the possibility to become multi-functional material because they have high optical properties produced from the advantages of organic dyes in addition to the high thermal stability based on the benefits of inorganic materials.

## 2. Experimental Section

### 2.1. Preparation of Nanohybrids and Nanocomposites

**Scheme 1:**
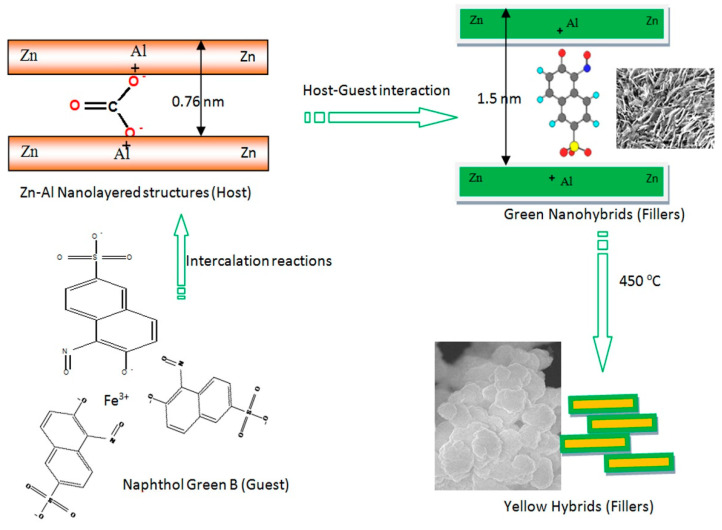
Formation of two kinds of nanohybrids (fillers) based on naphthol green B.

A series of green and yellow nanohybrids materials based on green dyes were produced by precipitating nanolayered structures of zinc and aluminum in presence of naphthol green B using urea hydrolysis and thermal treatment as shown in Scheme 1.

Two salts of zinc nitrate (0.046 M) and aluminum nitrate (0.018 M) were combined in presence of urea (0.5 M) in an aqueous medium. One gram of naphthol green B dyes was dissolved in the prepared aqueous solution. By heating the mixture at 90 °C for 14 h, the green powder of Zn-Al dyes nanohybrid was precipitated and separated through washing with distilled water. Different amounts of green dyes were used to produce five green nanohybrids as seen in Scheme 1. The obtained green powder was labeled NH-1, NH-2, NH-3, NH-4 and NH-5 according to the dyes amount 1, 2, 3, 4 and 5 g, respectively. The green nanohybrid NH-5 was thermally treated at 450 °C to become yellow nanohybrid NH-5-450 as shown in Scheme 1. In addition, the sample without dyes was prepared using the same method. This material was coded as ZA-1 and used as a reference material.

### 2.2. Preparation of Polymeric Nanocomposites

The pure poly (vinyl alcohol) 87–90% hydrolyzed, average molecular weight = 30,000–70,000, was obtained from Sigma-Aldrich. Two series of PVA nanocomposites were engineered using one of the prepared nanohybrids before and after calcination. The nanohybrids NH-5 and NH-5-450 were selected to be used as fillers for PVA because they have the high content of dyes confined inside their nanolayers. It was noted that the color of NH-5 was green while the powder of NH-5-450 has yellow color. 

In the first series of the PVA nanocomposites, 0.5 g of PVA was dissolved in 15 mL of deionized water with heating at 70 °C for 2 h under strong stirring to obtain a clear viscous solution. Similarly, 2 wt.% of NH-5 was dispersed and ultra-sonicated in deionized water. The suspended solution of NH-5 was mixed with the hot solution of PVA at 70 °C using magnetic stirring for obtaining homogeneous green solution of PVA. A thin green film was produced by pouring the green solution in a flat dish at room temperature. Then, the obtained films were coded as NCP-1, NCP-2 and NCP-3 according to the nanohybrid percentages 2, 10 and 20 wt.%, respectively.

For the second series of the PVA nanocomposites, the nanohybrid NH-5 was used as filler after calcination at 450 °C. By calcining the green powder of the nanohybrid, the color has changed to become yellow. This filler was coded by NH-5-450. The same procedure was used by adding the suspended solution of the filler NH-5-450 to the hot solution of PVA under magnetic stirring to obtain a homogeneous yellow solution. By pouring the yellow solution in a flat dish at room temperature, a thin yellow film was fabricated. The obtained films were coded as NCP-4, NCP-5 and NCP-6 according to the calcined nanohybrid percentages 2, 10 and 20 wt.%, respectively.

### 2.3. Characterization

Scanning electron microscopy JEOL-JSM-6330F (Japan) was used to determine the morphology of the prepared samples. Transmission electron microscopy was used by JEM 2100F (JEOL Company, Tokyo, Japan). Powder X-ray diffraction technique Bruker-AXS, Karlsruhe (Germany) has used Cu-K radiation source (λ = 0.154 nm) to determine the crystalline structures of the prepared nanomaterials. Thermogravimetric analyzer (Q500 TA Company, New Castle, PA, USA) was used for measuring thermal properties. Depending on the diffuse reflectance spectroscopy, the optical parameters of the prepared powder and films materials were measured using UV/VIS/NIR Shimadzu 3600 spectrophotometer (Shimadzu, Columbia, MD, USA) in the range 200–800 nm. 

## 3. Results and Discussion

### 3.1. Confinement of Naphthol Green B in Zn-Al Nanolayerd Structures

**Figure 1:**
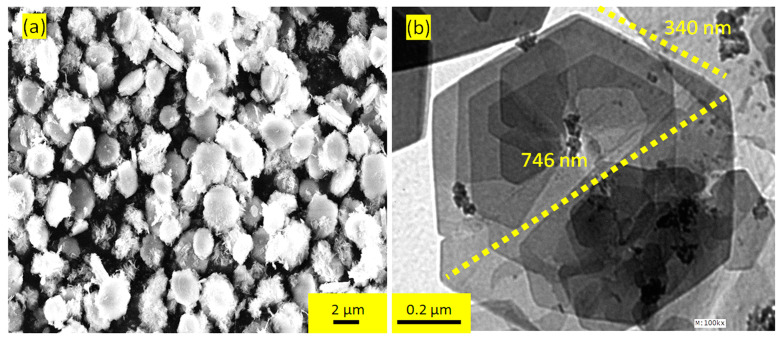
Images of the prepared sample ZA-1: (**a**) scanning electron microscopy and (**b**) transmission electron microscopy.

A plate-like structure is the conventional structure for synthetic LDHs and natural materials of LDHs (pyroaurite) exhibiting plates with dimensions in the range of millimeters [32]. In addition, hexagonal platy morphology was observed for the hydrotalcite crystals [33]. The morphology of the sample ZA-1 agrees with the conventional structure of synthetic and natural LDHs as shown in the SEM and TEM images in Figure 1. 

Figure 1a revealed an SEM image for ZA-1 indicating rounded plates. However, hexagonal nanoplates with a few hundred nanometers in width were observed in the TEM image of ZA-1 as shown in Figure 1b, which corresponds with the hydrotalcites structures because the TEM images show the individual plates of LDHs and SEM images and focus on aggregations or groups of plates. 

**Figure 2:**
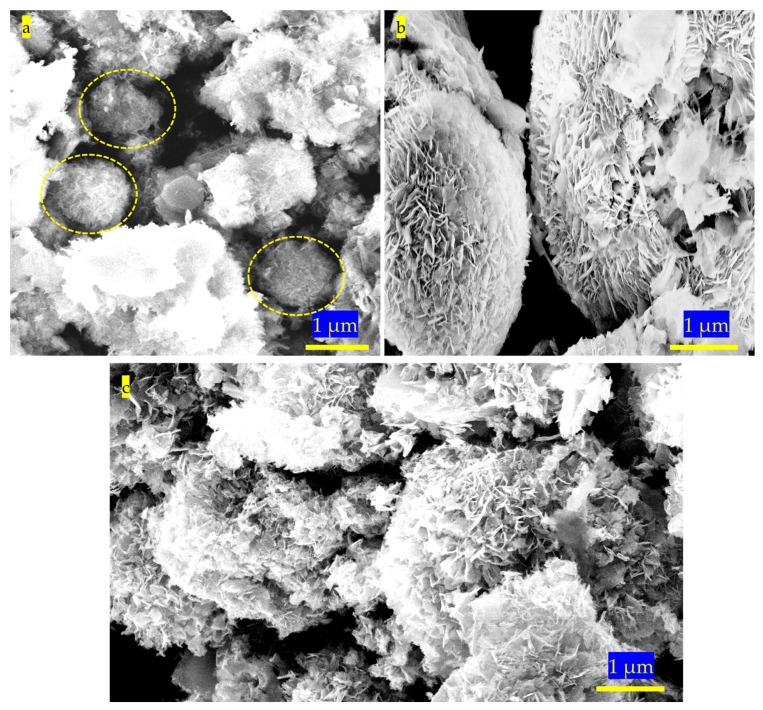
SEM images of the prepared nanohybrids: (**a**) NH-1, (**b**) NH-5 and (**c**) NH-3 (Circles indicate the aggregations of nanoplates).

These SEM and TEM images concluded that the sample ZA-1 belongs to the LDH group. By intercalating different percentages of green dyes, the strong aggregation of plates was observed as shown in Figure 2. The SEM image of NH-1 nanohybrid showed large cycles as seen in Figure 2a. These cycles consist of a large number of nanoplatelets that aggregated through self assembly behavior. This behavior was originated by generating a hydrophobic character for the nanoplatelets of Zn-Al LDH. The hydrophobic character was produced by bonding the aromatic molecules of the green dyes (two benzene rings) with the nanoplatelets of Zn-Al LDHs through host–guest interaction. By increasing the percentage of green dyes, Figure 2c indicated that the hydrophobic character enhanced as seen in the SEM image of NH-3. The self-assembly behavior of Zn-Al dyes was clearly observed through magnifying the SEM image of NH-5 in Figure 2b.

**Figure 3:**
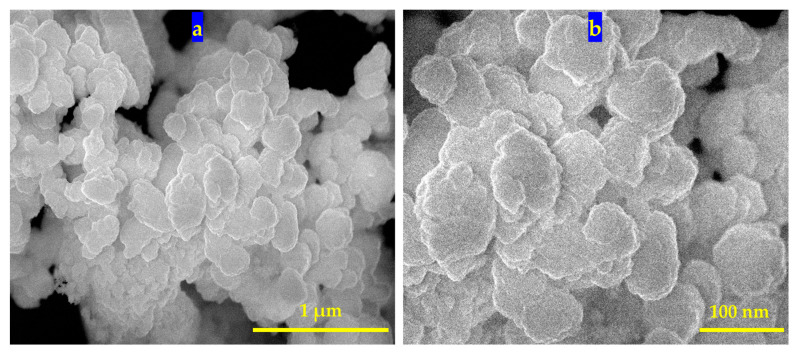
SEM images of the yellow nanohybrid NH-5-450: (**a**) at 1 µm and (**b**) at 100 nm.

Using thermal treatment of NH-5 at 450 °C, the green powder of the nanohybrid which was based on the naphthol green B converted to yellow powder because of the partial decomposition of the green dyes. Figure 3 showed SEM images of the new nanohybrid NH-5-450.

**Figure 4:**
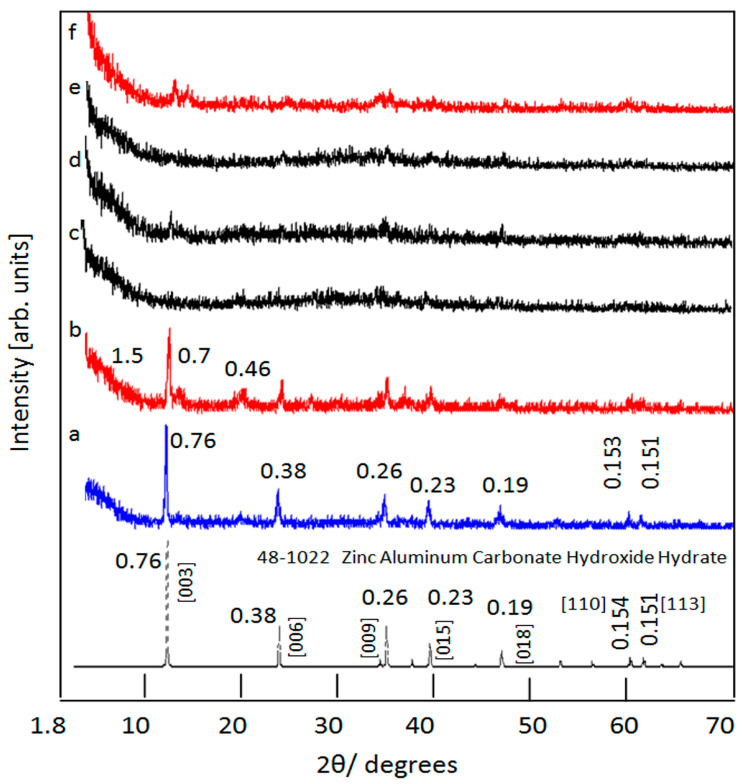
X-ray diffraction of: (a) ZA-1, (b) NH-1, (c) NH-2, (d) NH-3, (e) NH-4 and (f) NH-5.

SEM images indicated that the nanohybrid NH-5-450 has individual nanoplates as seen in Figure 3a. This means that the yellow nanohyrid has lost part of the hydrophobic character. In addition, Figure 3b showed that the size of the plate is 111 nm. Using X-ray diffraction, the nanolayered structure of ZA-1 was observed as shown in Figure 4a. The values of the basal peaks of planes hkl [003], [006] and [009] of ZA-1 showed an order arrangement of the packed stacks of brucite-like layers ordered along axis c; i.e., 0.76 nm = 2 × 0.38 nm = 3 × 0.26 nm.

According to the c dimension of the natural and synthetic hydrotalcite, which is assessed as three times the spacing for planes (003) and equals 2.31 nm [31], it nearly agrees with the calculated value for the prepared material ZA-1, i.e., 2.28 nm. Depending on the spacing for plane (110), the average distance between Zn-cation and Al-cation within the brucite-like layer, which is related to the lattice parameter ‘a’ of the prepared material and can be determined as two times d(110), could be calculated to be 2 × 0.153 nm = 0.306 nm agreeing with the reported data of LDHs [31]. These parameters confirmed that the sample ZA-1 has an LDH structure. 

The main peak of ZA-1 which was observed at 0.76 nm indicates the size and the orientation of the interlayered anions inside the layered structure of LDH. By comparing it with the layered structure of the synthetic Zn–Al LDH (JCPDS file No. 48-1022) and the natural hydrotalcite (JCPDS file No. 37-629), the main interlayered anions of the prepared material are carbonate anions as shown in Figure 4a.

The XRD pattern of the ZA-1 after intercalation with 3% of green dyes exhibited new weak reflections at lower 2θ in addition to the original basal spacing of LDH suggesting the formation of two phases: LDH and nanohybrid material as shown in Figure 4b.

The interlayer spacing of the LDH after the intercalation reaction with green dyes increased to be 1.5 nm. In addition, there are two other weak peaks at 0.7 nm and 0.46 nm. The successive reflections by basal planes, i.e., 1.5 nm ≈ 2 × 0.7 nm ≈ 3 × 0.45 nm, indicated a layered structure for the prepared nanohybrid. The reflection of (110) became unclear. In addition, the intercalation of organic species decreased the order arrangement of the layered structure. By increasing the percentage of green dyes through intercalation reactions, the layered structure of LDHs disappeared as shown in Figure 4c–f. This means that the presence of aromatic molecules among the layers of nanohybrid led to distortion of the nanolayers because of the steric hindrance and repulsion of the benzene rings inside the interlayered space. This means that the absence of reflections corresponding to the nanolayered structure of LDH after organic modification is due to the disappearance of coherent conditions in different directions (lack of repeated units).

**Figure 5:**
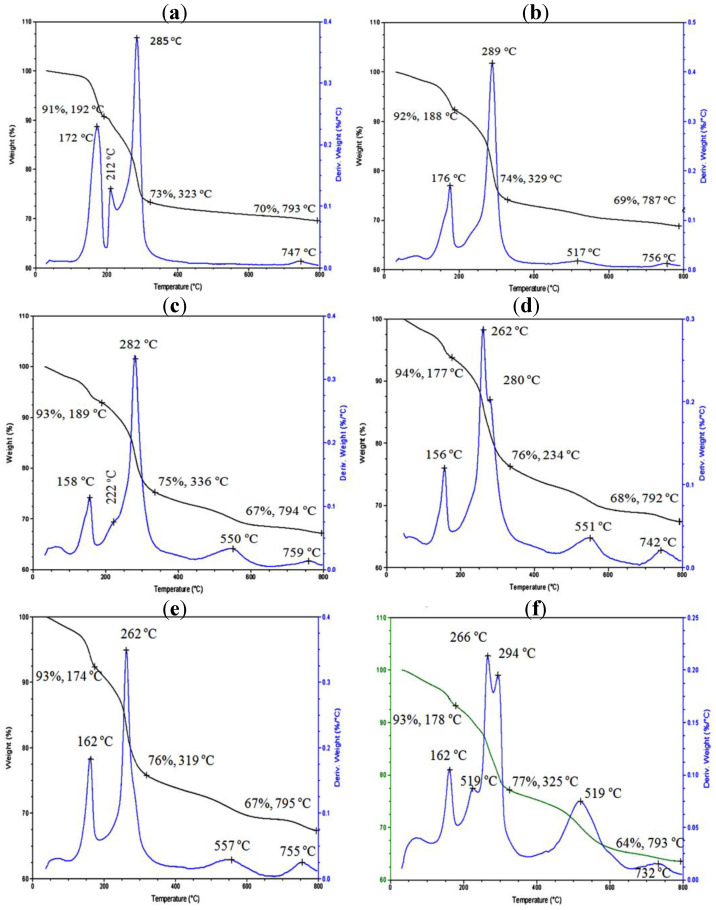
TG and DTG curves of (**a**) ZA-1, (**b**) NH-1, (**c**) NH-2, (**d**) NH-3, (**e**) NH-4 and (**f**) NH-5 (the black and green lines are TG curves and the blue lines are DTG curves).

The presence of organic molecules inside LDH structures and the formation of organic-inorganic nanohybrids were confirmed by the data of thermal analyses (TG and DTG). Thermal characteristics of the sample ZA-1 were measured by the diagrams of both TG and DTG as shown in Figure 5a. The weight of ZA-1 was mainly lost through two stages. In the first stage, 9 wt.% was lost at 192 °C corresponding to the desorption of surface and intercalated water. The second stage was achieved at 323 °C to lose 18 wt.% agreeing with the removal of hydroxyl groups of LDHs and decomposition of anions. In the DTG diagram, three peaks were observed at 172 °C, 212 °C and 285 °C as shown in Figure 5a. At first peak, the removal of both surface and interlayered water happened at 172 °C agreeing with the TG data. The other two peaks belonging to the removal of hydroxyl groups of LDHs and decomposition of anions occurred at 212 °C and 285 °C matching with the second stage of the TG diagram.

Thermal characteristics of Zn–Al dyes nanohybrids were observed in Figure 5b–f indicating three stages for weight losses. The first and second weight losses in all samples are due to the removal of water molecules and decomposition of carbonate anions, respectively. The third weight loss confirms the intercalation of aromatic rings of the green dyes inside LDH structures.

In the case of the sample NH-1, the DTG diagram showed a new peak at 517 °C agreeing with the third weight loss in the TG diagram as seen in Figure 5b. By increasing the percentage of green dyes during the intercalation reactions of the samples from NH-1 to NH-5, the third weight loss in TG curves increased from 5% to 13%, respectively. In addition, the DTG diagrams showed that the intensity of the peak at 519 °C increased arriving at a maximum at sample NH-5 as shown in Figure 5.

Compared with the parent LDH (ZA-1), the content of the interlayered water of the nanohybrids decreased from 9% to 6%. In addition, new peaks were observed in the TG diagram at the temperature range 262–266 °C and 517–557 °C for the prepared nanohybrids NH3-NH5 confirming the intercalation of organic species instead of carbonate anions.

### 3.2. Colored Polymeric Nanocomposites

According to the above results, the sample NH-5 considers the most suitable nanohybrid to be used as a filler for building colored polymeric nanocomposites because it has organic–inorganic nanostructures and contains 13 wt.% of green dyes. 

Therefore, this nanohybrid was used as a filler for poly vinyl alcohol through two different ways. The first way depends on intercalation of the nanohybrid before calcination. In the other way, the nanohybrid has been used after calcination at 450 °C.

**Figure 6:**
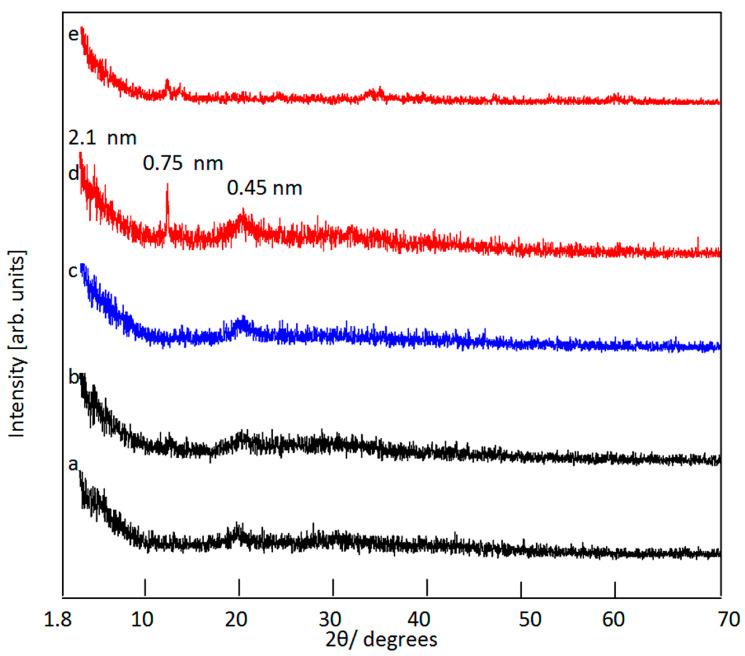
X-ray diffraction of the PVA nanocomposites: (a) pure PVA, (b) the filler NH-5, (c) NC-1, (d) NC-2 and (e) NC-3.

By using the first way, three polymeric nanocomposites were produced using different percentages of the nanohybrid. NCP-1, NCP-2 and NCP-3 were prepared by intercalating 2 wt.%, 10 wt.% and 20 wt.% of the nanohybrid inside PVA. The XRD pattern of the pure PVA was observed in Figure 6a revealing a weak peak at 2θ = 19° (d-spacing = 0.45 nm) indicating the characteristic peak of the plane (101) of PVA. In addition, this peak indicates the intermolecular hydrogen bonds that connect between the chains of PVA through hydroxyl groups [34,35]. Furthermore, Figure 6c–e showed the XRD patterns of the nanocomposites NCP-1, NCP-2 and NCP-3. For the nanocomposite NCP-1, Figure 6c showed amorphous structure indicating that the low percentage of the nanohybrid (2 wt.%) is homogeneously dispersed inside the matrix of PVA. The disappearance of the diffraction maximum of the nanohybrids may be the result of the subsequent increase in the interlayer periodicity and the shift of the maximum to the low-angle region beyond the range of the diffractometer used.

In the case of high percentages of the nanohybrid, new nanolayered structures were observed for the polymeric nanocomposite NCP-2 and NCP-3 as shown in Figure 6d,e. X-ray diffraction patterns of NCP-2 and NCP-3 showed weak peak at 2.1 nm and clear peak at 0.75 nm in addition to the peak of PVA. This means that the polymer chains of PVA have intercalated among the nanolayers of the nanohybrids and expanded the inlayered spacing of the nanocomposites to become 2.1 nm. Compared with the X-ray patterns of the nanohybrid and the parent PVA, the intercalation process increased the crystalline structure of PVA creating a new phase of polymeric nanocomposite Zn-Al-dyes-PVA.

**Figure 7:**
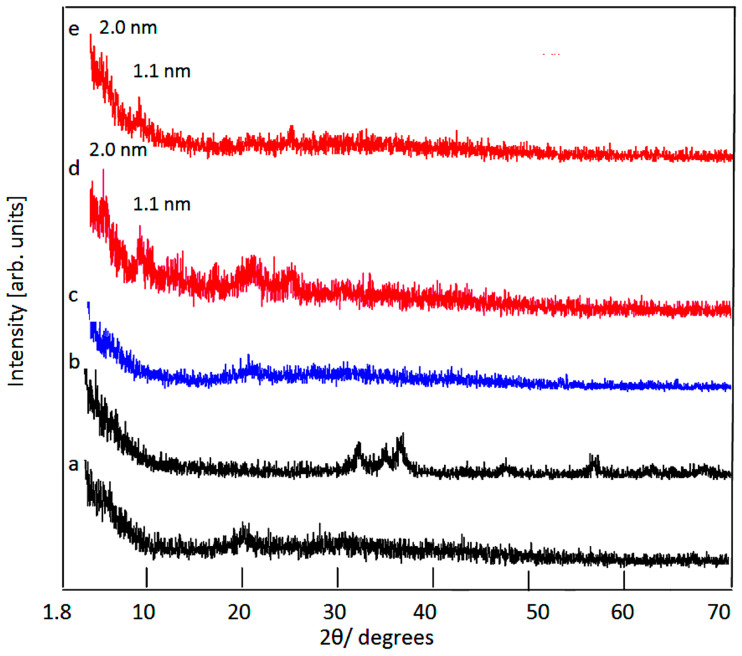
X-ray diffraction of the PVA nanocomposites: (a) pure PVA, (b) the filler NH-5-450, (c) NC-4, (d) NC-5, and (e) NC-6.

In the case of using the second way of the intercalation process, another three samples of polymeric nanocomposites NCP-4, NCP-5 and NCP-6 were prepared through inserting 2 wt.%, 10 wt.% and 20 wt.% of the calcined nanohybrid inside PVA. The X-ray diffraction of the calcined nanohybrid showed that it has a zinc oxide structure agreeing with wurtzite crystals (JCPDS 36-1451) as shown in Figure 7b. This means that the calcined nanohybrid has a zinc oxide structure doping with aluminum and the products that produced from the decomposition of green dyes. By intercalating the low percentage of the calcined nanohybrid, the peaks of zinc oxide disappeared as shown in Figure 7c. An X-ray diffraction pattern of NCP-4 showed only weak peak at 0.45 nm indicating that the nanohybrid layers are completely and uniformly dispersed in the structure of PVA. By increasing the percentage of the calcined nanohybrid, new weak peaks were observed in the XRD patterns of both NCP-5 and NCP-6 as seen in Figure 7d,e. Figure 7d showed two peaks at low two theta with d-spacing at 2.0 nm and 1.1 nm. This means that the nanolayers of the nanohybrid were rearranged to build the nanolayered structure again in the presence of polymer chains depending on the phenomenon of memory effect of LDH structure. This phenomenon is familiar in the LDH structure. After heating the LDH at a temperature of 450 °C for a few hours, the nanolayered structures of LDHs are converted into mixed oxides, but still have memory for their own structure [36,37]. These mixed oxides, after dispersion in an aqueous solution containing preferable anionic moieties followed by some degree of aging, revert to the original layered structures of LDHs.

### 3.3. Thermal Stability

**Figure 8:**
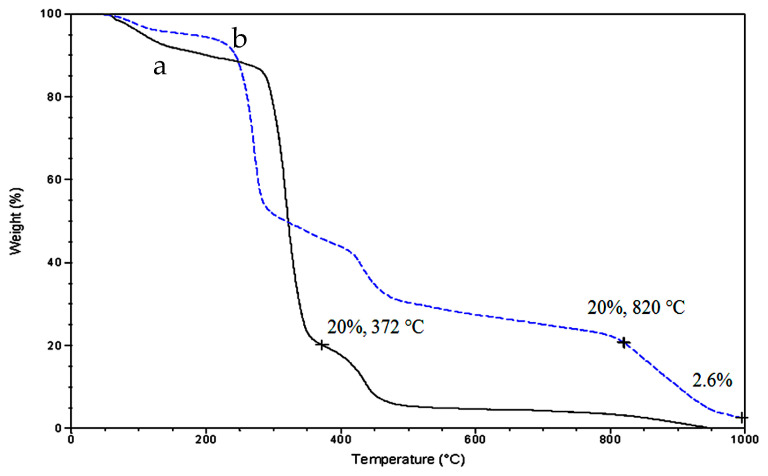
TG Curves of (a) pure PVA and (b) the nanocomposite NCP-3.

**Figure 9:**
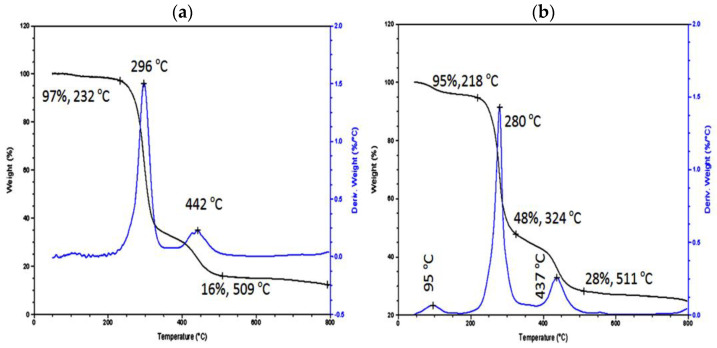
DTG and TG Curves of: (**a**) NCP-1 and (**b**) NCP-2 (the black lines are TG curves and the blue lines are DTG curves).

**Figure 10:**
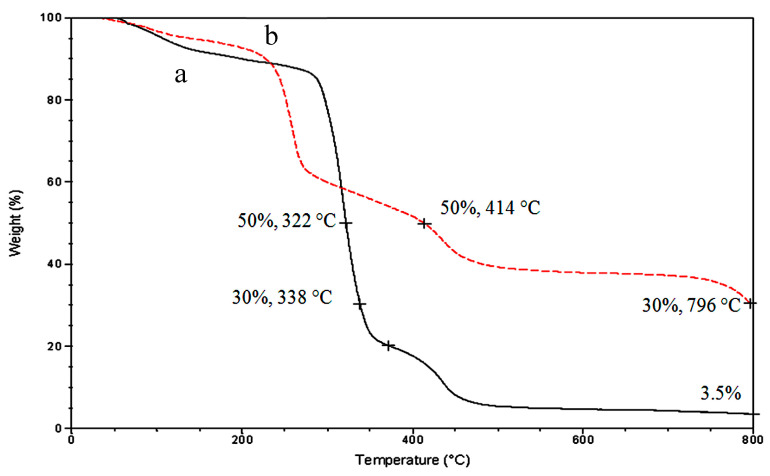
TG Curves of (a) pure PVA and (b) the nanocomposite NCP-6.

**Figure 11:**
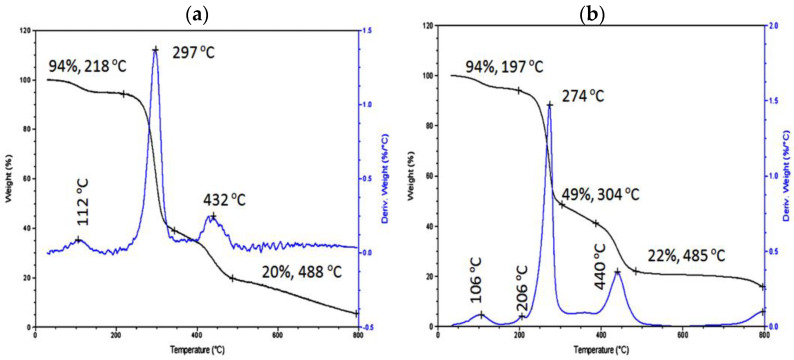
DTG and TG Curves of: (**a**) NCP-4 and (**b**) NCP-5 (the black lines are TG curves and the blue lines are DTG curves).

The measurements of TGA and DTG have been used for studying the effect of green and yellow nanohybrids on the thermal stability of PVA through building nanocomposites as shown in Figure 8, Figure 9, Figure 10 and Figure 11.

Figure 8 indicates the different stages of thermal decay of pure PVA and the nanocomposite NCP-3. Figure 8a showed that the main weight of the pure PVA (80%) was lost at 372 °C through two stages. In case of the nanocomposite NCP-3, the TG curve showed that it has four stages for losing 80% of NCP-3 at 820 °C. 

It is noted that the temperature corresponding to the first loss of 5% (T0.05) of the pure PVA is 105 °C. This temperature was shifted to a higher value at 175 °C for the nanocomposite NCP-3. The final residue of NCP-3 which was obtained at 1000 °C was 2.6%, while there was no residue for the pure PVA. These data confirmed the positive effect of nanohybrids on the thermal stability of the PVA. 

Similar results were observed for the nanocomposites NCP-1 and NCP-2. Figure 9 showed that the temperature of the initial 5% weight loss of both NCP-1 and NCP-2 occurred at 250 °C and 210 °C. In the same trend, the nanocomposites NCP-1 and NCP-2 have lost 80% of their weight at higher temperatures (457 °C and above 800 °C) compared with the pure PVA. 

In the case of the second series of nanocomposites NCP-6, NCP-5 and NCP-4, the thermal stability became higher than the pure PVA agreeing with the first series of nanocomposites. Figure 10 showed that 50% of the nanocomposite NCP-6 was lost at 414 °C while for the pure PVA this was observed at 322 °C. For a weight loss of 70%, Figure 10 showed similar behavior because this was achieved at 796 °C for NCP-6 while it was observed at a lower degree for the pure PVA at 338 °C. Figure 11 indicated that the nanocomposites NCP-4 and NCP-5 have higher thermal stability than the pure PVA.

The decomposition of the chains of polymer starts at their ends or the weak bonds through formation of free radicals. After that, the degradation process continues by transferring to the adjacent chains via interchain reactions. In the prepared nanocomposites, the presence of the inorganic nanolayers slowed down the mobility of the chains of the polymer because of the hydrogen bonds which generated between the PVA chains and nanohybrid materials to improve the thermal stability of the polymer [38].

### 3.4. Optical Properties

**Figure 12:**
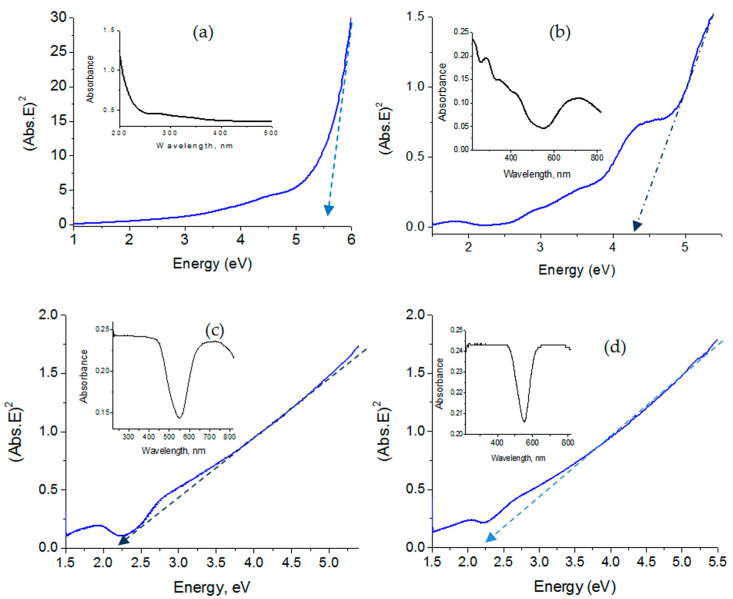
UV–Vis absorbance and band gap energy of: (**a**) pure PVA, (**b**) NCP-1, (**c**) NCP-2 and (**d**) NCP-3 (the black lines are absorbance curves, the blue lines are the band gap curves and the arrows indicate the band gap energy).

It is known that poly (vinyl alcohol) has no clear optical behavior. In order to improve its optical properties, six samples of its nanocomposites were prepared and studied by the important details about their absorbance and band gaps that were determined through the UV–Vis absorption spectra. Figure 12a showed the UV–Vis absorbance and band gap of the pure PVA. Two weak absorption peaks are recorded at 200 nm and 300 nm indicating that it has a wide band gap as shown in Figure 12a.

By plotting the incident photon energy (E) and the absorbance coefficient of the materials (Abs), the energy band gap (E_g_) was assessed through the following procedure [39]:

(Abs.E)^2^ = constant (E − E_g_)

where E = hc/λ; the values (h and c) are the Planck’s constant and speed of light. The value (Abs) means the absorption coefficient. 

In order to determine the band gap energy, both (Abs.E)^2^ and (E) were plotted. When the value (Abs.E)^2^ equals zero, the energy matches with the optical band gap. This is achieved through extending the straight line to the (E) axis, as shown in Figure 12a. Figure 12a indicated that the pure PVA has a band gap energy at 5.5 eV. 

In the case of the nanocomposite NCP-1, Figure 12b (inset) revealed that the optical properties of PVA improved through shifting the absorption edge of PVA to 800 nm. In addition, the absorbance range become broader in the UV region to cover the range between 500 nm and 200 nm as shown in Figure 12b (inset). This means that the low content of nanohybrid created new optical sites inside the PVA matrix to become active in the visible and UV regions after building the nanocomposite. These results were confirmed by calculating the band gap energy. Figure 12b showed that the nanocomposite NCP-1 has a band gap energy at 4.3 eV. 

By increasing the content of the nanohybrid inside PVA, more improvement happened for the polymer as shown in the spectra of both NCP-2 and NCP-3. Figure 12c (inset) showed the absorption spectra and band gaps of both NCP-2 and NCP-3. Clear absorption was observed in the UV region in the range of 450 nm to 200 nm. Furthermore, in the visible region, strong absorption was observed in the range of 600–800 nm. In addition, the band gap decreased to be 2.2 eV for both NCP-2 and NCP-3 as seen in Figure 12c,d.

**Figure 13:**
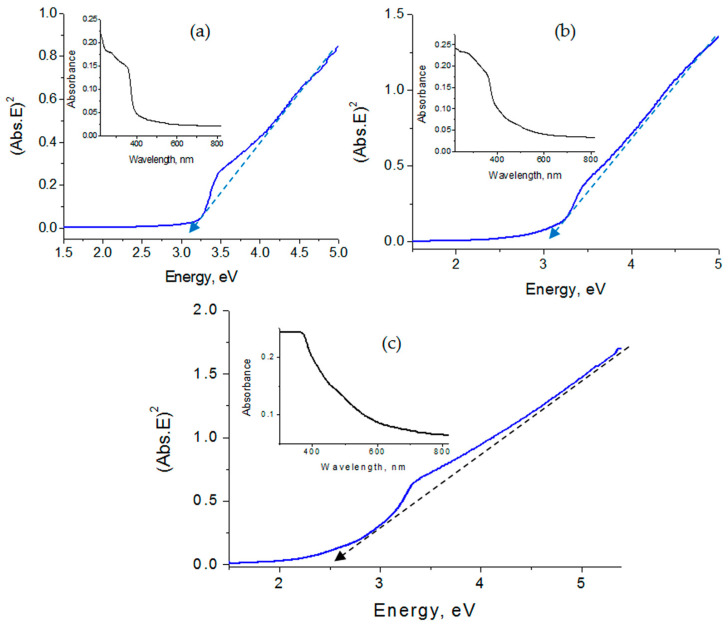
UV–Vis absorbance and band gap energy of: (**a**) NCP-4, (**b**) NCP-5 and (**c**) NCP-6 (the black lines are absorbance curves, the blue lines are the band gap curves and the arrows indicate the band gap energy).

In the second series of nanocomposites, another trend for improving the optical behavior of PVA was observed for the samples NCP-4, NCP-5 and NCP-6. In the sample NCP-4, the absorption edge of PVA shifted to a higher wavelength of 400 nm as shown in Figure 13a (inset). This means that the PVA became active in the UV region. Figure 13a showed that the band gap of NCP-4 was observed at 3 eV. In the same trend, the sample NCP-5 showed more shifting for UV absorption to arrive at 500 nm indicating that it has a band gap at 3 eV as seen in Figure 13b.

By increasing the content of the nanohybrid in the second series, Figure 13c showed continuous shifting for the absorption edge of NCP-6 to be at 700 nm in the visible region. This means that NCP-6 became active in the visible region because its band gap decreased to become 2.5 eV.

This lowering of the band gap of PVA can be explained through the formation of new optical sites inside the polymer structure because of the presence of the optical active dyes combined with optical inorganic nanolayers. The band gap energy of the pure PVA (5.5 eV) is very wide because the large gap between the HOMO and LUMO bands of PVA. For the prepared nanocomposites, the presence of the nanolayers of the Al-ZnO, which were coated with molecules of dyes, created trap levels between the HOMO and LUMO bands of PVA producing dangling orbitals. At the same time, this increased the band tails in the polymeric nanocomposites. Therefore, the band gap strongly decreased to become 2.5 eV. This explanation agrees with many previous studies [40,41,42,43,44,45].

## 4. Discussion

It is known that poly vinyl alcohol is a colorless polymer and has absorbance bands only in the deep UV range as shown in Figure 12a. In the visible region, there are no absorbance bands, so it is considered optically inactive. 

Recently, Alrowaili et al. [40] have used organic carmine dye to increase the optical properties of PVA. They reported that the bandgap of PVA decreased from 5.16 to 1.65 eV as the organic dye percentage increased. Aziz et al. [41] have used inorganic species for improving the optical parameters of PVA through decreasing the band gap energy of PVA/zinc oxide nanocomposite to reach 2.80 eV.

**Figure 14:**
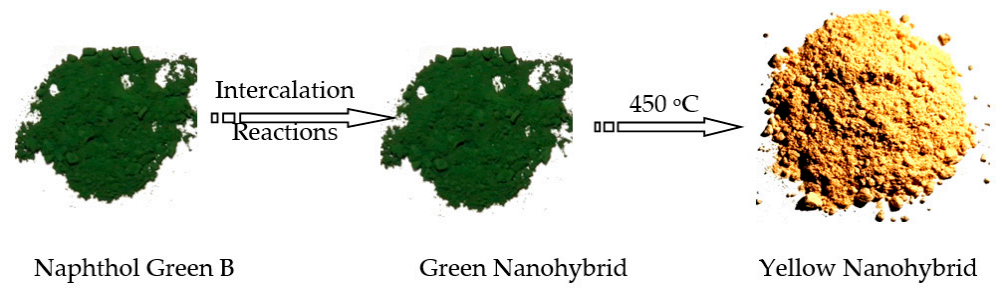
Images of the different colors of the powder of nanohybrid.

Therefore, in the current study, both organic dyes and inorganic species have combined through nanohybrid structures that were formed through confining naphthol green B among nanolayers of Zn-Al layered structures to produce green nanohybrids as shown in Scheme 1. By thermal treatment of the prepared green nanohybrids, the green color of the nanohybrid transformed to a yellow color indicating partial oxidation of the confined green dyes and producing new yellow nanohybrids as seen in Figure 14.

**Figure 15:**
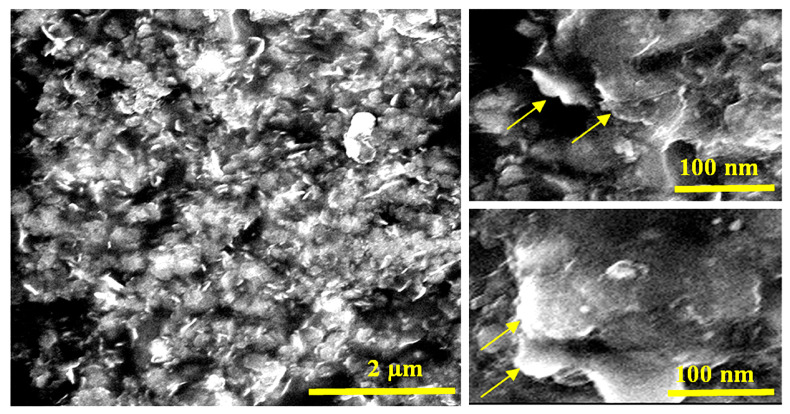
SEM images of the polymeric nanocomposite NCP-2 (the arrows indicate nanoplates).

**Figure 16:**
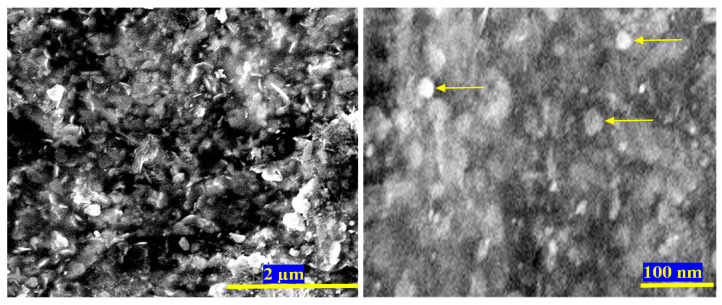
SEM images of the polymeric nanocomposite NCP-5 (the arrows indicate very fine nanoplates).

It is known that PVA consists of an organic part and a large amount of hydroxyl groups. At the same time, the nanohybrids are composed of organic dyes and hydroxyl groups in addition to inorganic nanolayers. Therefore, the combination between the nanohybrids and PVA can create a large amount of hydrogen bonds which are formed from the hydroxyl groups. In addition, the organic part of PVA is compatible with the organic dyes of the nanohybrids. These factors led to the formation of homogeneous polymeric nanocomposites as shown in the morphology of the products shown in Figure 15 and Figure 16. By inserting the green nanohybrids, the inorganic Zn-Al nanolayers, which attached with organic molecules of the dye, were homogenously dispersed among the hydrocarbon chains of PVA through an exfoliation process as seen in SEM images in Figure 15. In addition to the compatibility between the hydrophobic character of both the organic dye and the hydrocarbon chain of PVA, the hydrogen bonds were formed between the hydroxyl groups of the Zn-Al nanolayers and the hydroxyl groups of the polymer to produce green and transparent polymeric nanocomposites as shown in Figure 15.

X-ray diffraction confirmed the arrangement of the inorganic Zn-Al nanolayers between the PVA chains through observing the crystalline structure of polymeric nanocomposite NCP-2 although the PVA structure is non-crystalline as shown in Figure 6. In the case of using the yellow nanohybrids, the zinc oxide nanoplatelets, which were modified by aluminum and organic species, were well dispersed inside the matrix of PVA as seen in SEM images in Figure 16. In addition, X-ray diffraction showed that the nanolayered structures were rearranged through the memory effect of Zn-Al LDHs after combining the yellow nanohybrids with PVA as shown in Figure 7.

According to these polymeric nanocomposites, PVA nanocomposites became optically active because they have clear absorbance bands in UV and visible regions. Furthermore, the band gap energy decreased from 5.5 eV to reach 2.2 eV and 2.5 eV after combining with the green nanohybrids and the yellow nanohybrids, respectively. The absorbance in the visible region can be explained through the transitions from the ground state S_o_ of the dye to the excited state S_1_ [42,43]. In addition, the narrowing in the band gap energy is due to forming trap levels between HOMO and LUMO bands of PVA producing dangling orbitals, increasing the band tails in the polymeric nanocomposites [44,45].

In addition, these polymeric nanocomposites improved the thermal stability of poly vinyl alcohol as shown in Table 1. Where, the green and yellow nanohybrids reduced the mobility of the chains of the polymer through the formation of hydrogen bonds with the chains of PVA. In this way, the free radicals mechanism for degradation of the chains of polymer, which starts at their ends or the weak bonds, did not continue to the adjacent chains [38]. Therefore, the degradation temperature of the nanocomposites shifted to higher values as shown in Table 1. It can be concluded that the nanohybrids have a dual function for improving the thermal stability of PVA with generating new optical behavior.

## 5. Conclusions

In the current study, the confinement of the naphthol green B in the nanoscale inside a two-dimensional Zn-Al nanostructure was used to achieve a dual effect for poly vinyl alcohol. The first effect was the conversion of non-optical-active PVA to become optical active polymeric nanocomposites in a wide range of wavelengths. In the second effect, the high thermal stability was observed for these polymeric nanocomposites. This dual effect was clear after inserting the green nanohybrids which were used as prepared or the yellow nanohybrids which were produced after thermal treatment inside the matrix of PVA. In the case of using the green nanohybrids, strong narrowing of the band gap energy of PVA was observed from 5.5 eV to 2.2 eV. By using the yellow nanohybrids, the energy band gap decreased to become 2.5 eV. The other effect of the confined dye among the inorganic nanolayers was conversion of the hydrophilic character of inorganic species to hydrophobic behavior to be compatible with the hydrocarbon chains of PVA. This effect led to well dispersing for the inorganic nanolayers inside the PVA matrix and improving the thermal properties of the prepared polymeric nanocomposites. Where, the prepared nanocomposites have lost 80% of their weight at higher temperatures (from 457 °C to above 800 °C) comparing with the pure PVA (372 °C) because the presence of the inorganic nanolayers decreased the thermal degradation process through reducing the mobility of the polymer chains. Finally, these findings concluded that the prepared PVA nanocomposites can be used for optical applications in the UV and visible regions such as UV absorbers or solar cells.

## Figures and Tables

**Table 1:** Thermogravimetric data for the pure poly vinyl alcohol and their nanocomposites.

Samples	Fillers	Fillers %	T_0.05_ (5%) (°C)	T_0.80_ (80%) (°C)
Pure PVA	No	0	105	372
NCP-3	Green Nanohybrid	20	175	820
NCP-2	Green Nanohybrid	10	210	Above 800
NCP-1	Green Nanohybrid	2	250	457
NCP-6	Yellow Nanohybrid	20	136	Above 800
NCP-5	Yellow Nanohybrid	10	160	691
NCP-4	Yellow Nanohybrid	2	174	488

## Data Availability

Data available in a publicly accessible repository.
